# Efficacy of Topical Vitamin C Application on Healing After Gingival Depigmentation by Scalpel: A Case Series

**DOI:** 10.7759/cureus.48417

**Published:** 2023-11-06

**Authors:** Avreet Sandhu, Divya Jyoti, Harmesh Sharma, Tanvi Phull, Navjot S Khurana, Jaspreet K Tiwana

**Affiliations:** 1 Department of Orthodontics and Dentofacial Orthopaedics, Luxmi Bai Institute of Dental Science and Hospital, Patiala, IND; 2 Department of Oral Health Sciences (Periodontics), Post Graduate Institute of Medical Education and Research (PGIMER) Chandigarh (SB), Chandigarh, IND; 3 Department of Periodontology, Punjab Government Dental College and Hospital, Amritsar, IND; 4 Department of Oral and Maxilofacial Surgery, Gian Sagar Dental College, Patiala, IND; 5 Department of Conservative Dentistry and Endodontics, Government Dental College, Patiala, Patiala, IND; 6 Department of Pediatric Dentistry, Government Dental College, Patiala, Patiala, IND

**Keywords:** anterior aesthetics, scalpel, vitamin - c, periodontal surgery, gingival melanin pigmentation, gingival hyperpigmentation, gingiva

## Abstract

Every person expresses their blissful moments through a smile. The elegance of the smile depends majorly on the colour of the gingiva. One of the factors that determines the colour of the gingiva is the amount of melanin pigment in the gingival epithelium. The intensity of melanin pigmentation differs from one person to another, and it is prevalent among all ethnicities. Most people are aesthetically concerned and widely prefer pink gingiva, leading to a demand for gingival depigmentation.

There are various suggested treatment modalities for gingival depigmentation, like scalpel, electrosurgery, LASER, cryosurgery, etc. However, the recurrence of pigmentation is common in the depigmentation procedure. The current study attempts to determine the post-operative healing of gingival tissues following split-thickness epithelial excision with and without topical application of Vitamin C (ascorbic acid).

## Introduction

The curve of a smile has the power to make everything go straight. When it comes to aesthetics, gingival colour is crucial. The thickness of the gingival epithelium, vascularity, degree of keratinisation and amount of melanin pigments deposited on the superficial layer of the gingival epithelium determine the colour of the gingiva. The colour and extent of pigmentation of the gingiva vary among different individuals and different races [1].

Gingival hyperpigmentation is a condition wherein there is excessive deposition of melanin pigment, which is produced by the melanocytes of the gingiva. The melanocytic activity depends upon their enzyme tyrosinase, which later gets condensed into membrane-bound vesicles called pre-melanosomes, which are considered to be the precursors of melanocytes [2]. These intense pigmentations can either be physiological or pathological. If hyperpigmentation is observed as a genetic trait, then most likely it is physiological. Whereas, pathological pigmentation could either be due to exogenous causes such as drugs, tobacco smoking, amalgam tattoos, or heavy metal deposition, or endogenous due to endocrine disorders, syndromes, chronic irritation, or neoplasia [3]. The removal or reduction of this disagreeable appearance of gingival pigmentation comes under a periodontal plastic surgical procedure named gingival depigmentation, wherein the superficial layer of melanin-containing gingiva is removed. People who are aesthetically concerned usually demand gingival depigmentation.

Various depigmentation techniques have been described in the literature, which can be broadly classified into chemical and surgical methods [4]. Chemical agents such as alcohols, phenols, and ascorbic acid are used. Surgical methods comprise conventional techniques, electrosurgery, lasers, cryosurgery, and radiosurgery. Conventional techniques include gingival abrasion, split-thickness epithelial excision/scalpel surgical technique/surgical stripping, free gingival grafting, and acellular dermal matrix allograft (ADMA).

The human body is unable to synthesise vitamin C, which is also called ascorbic acid [5]. On introduction into the target tissue, vitamin C prevents the adhesion of melanocytes to the adjacent keratinocytes by scavenging the calcium and copper content, which are necessary for cellular binding. This failure of melanocytes to adhere to adjacent keratinocytes prevents the activation of melanocytes, thereby reducing the production of melanin [6]. Also, vitamin C is an antioxidant that plays an important role in scavenging free radicals and aids in the accelerated healing of tissues [7]. This case report describes two cases of gingival depigmentation using a scalpel surgical technique with the adjunctive application of topical vitamin C ointment.

## Case presentation

Two male patients aged 22 and 25 years, respectively, reported to the Department of Periodontics, Punjab Government Dental College, Amritsar, India, for routine oral prophylaxis. Upon intraoral inspection, it was found that the gingiva had a diffuse blackish pigmentation, which was very noticeable in the upper and lower anterior regions (on the labial surface of the gingiva) in both cases. Patients were informed of the unappealing gingival pigmentation and the various aesthetic treatment options available to them.

**Figure 1:**
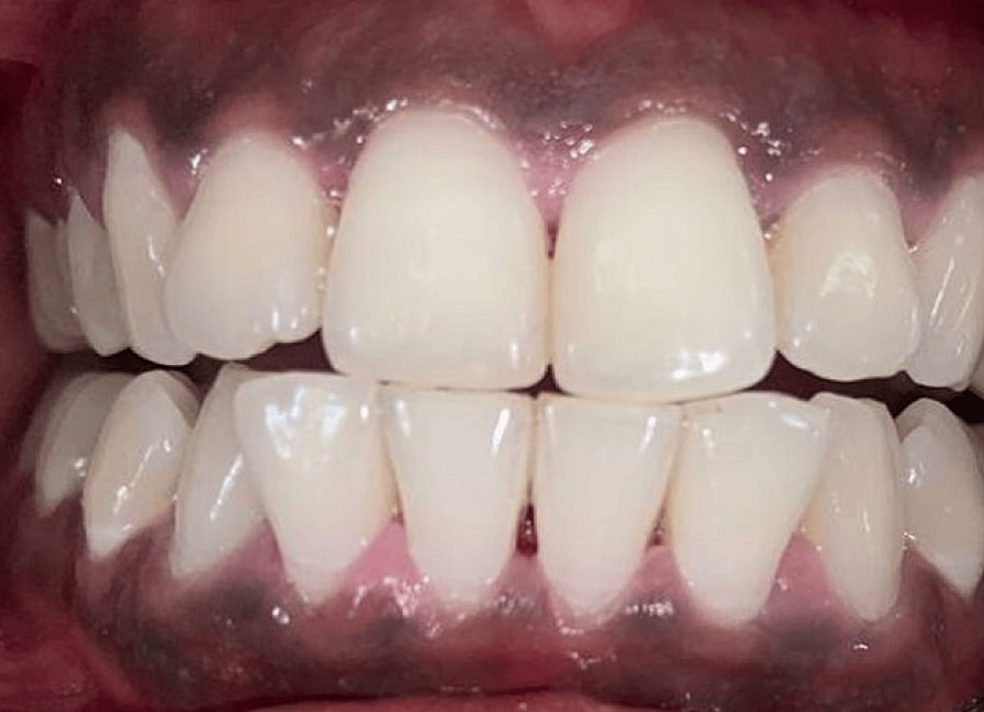
Case 1 - pre-operative image (DOPI score-3). Oral inspection showed noticeable bilateral melanin pigmentation. DOPI: Dummett-Gupta Oral Pigmentation Index.

**Figure 2:**
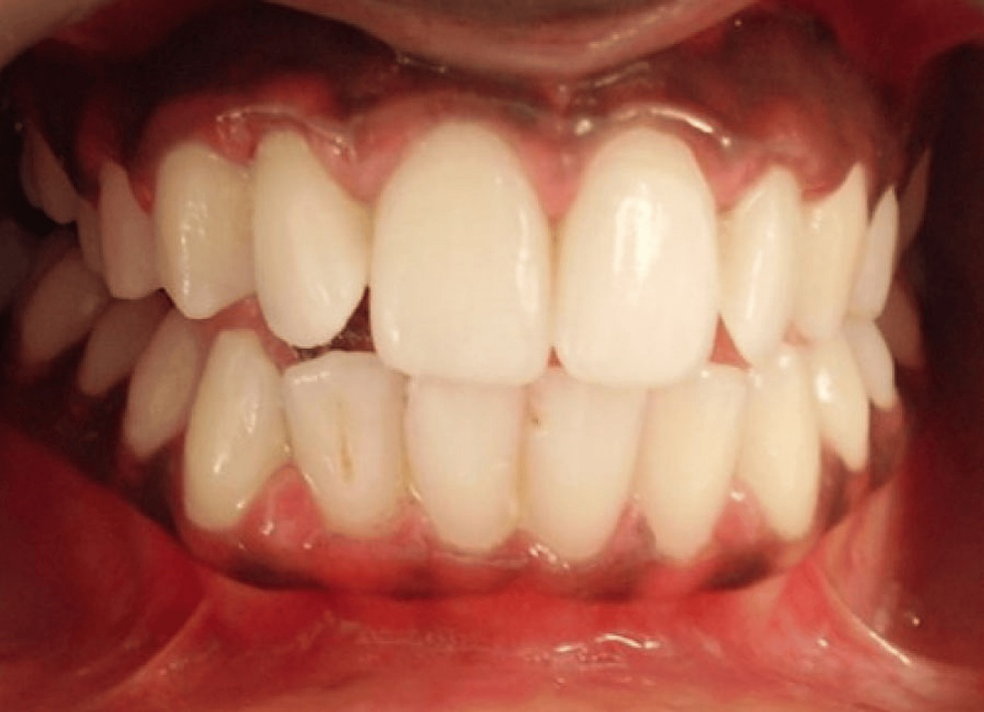
Case 2 - pre-operative image (DOPI score-2). Oral inspection showed noticeable bilateral melanin pigmentation.

The patients gave a history of congenital blackish discoloration of the gingiva, suggestive of physiologic gingival melanin pigmentation. Oral inspection showed noticeable bilateral melanin pigmentation, as shown in Figures 1-2. Oral hygiene was well maintained by both patients. There was no associated medical history, and the patients were systemically healthy. There was no history of tobacco or alcohol consumption. There were no contraindications for the surgical procedure to be performed. We explained various management options for the same. The patients decided to get the depigmentation operation done. The patients were aesthetically concerned, and, on their demand, a gingival de-epithelization procedure was planned.

It was intended to de-epithelize the gingiva using a scalpel (BP handle 3, blade no. 15). Procedures for depigmentation were scheduled upon patient permission. Phase I therapy, including oral prophylaxis, ultrasonic scaling, and polishing, was performed. Once the inflammation had subsided, depigmentation surgery was scheduled. Local infiltration with Lignocaine HCl containing 1:80,000 adrenaline was administered to the patient. From canine to canine, split-thickness epithelial excision was performed. Using a 15-number blade held parallel to the long axis of the teeth, a topical anaesthetic (lidocaine 2% with 1:80,000 epinephrine) was administered before the superficial layer of the gingiva was meticulously scraped. In order to prevent post-operative gingival pitting, minimal force or pressure was applied. A pressure pack made of sterile gauze was used to stop the bleeding. For the first patient, vitamin C (commercially known as Enshine Cream 15 g) was topically applied; a periodontal pack was used to cover the surgical sites; and post-operative instructions were provided. The second patient did not receive a vitamin C application. The management of pain involves the prescription of analgesics.

**Figure 3:**
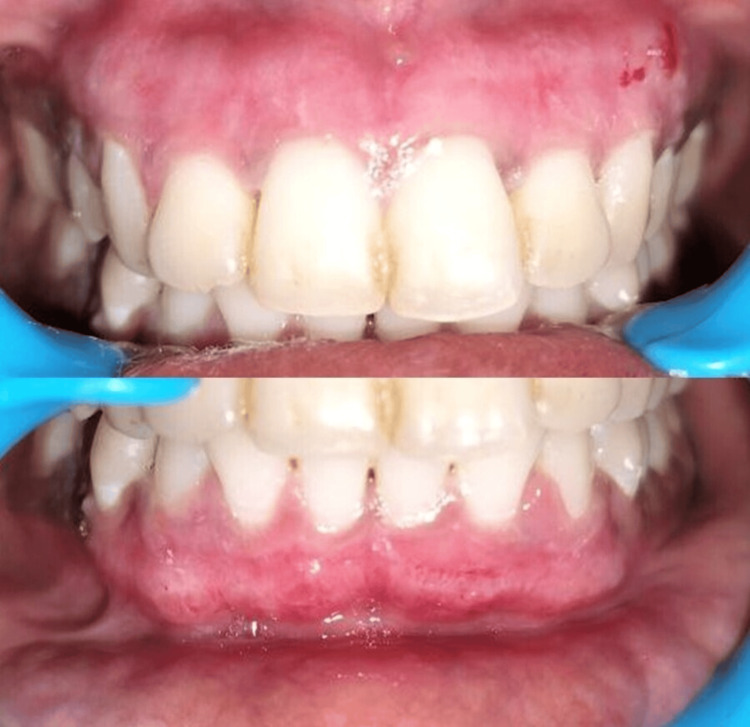
Case 1 - post-operative image. The recovery went smoothly with no post-surgical complications encountered. Vitamin C application showed satisfactory healing.

**Figure 4:**
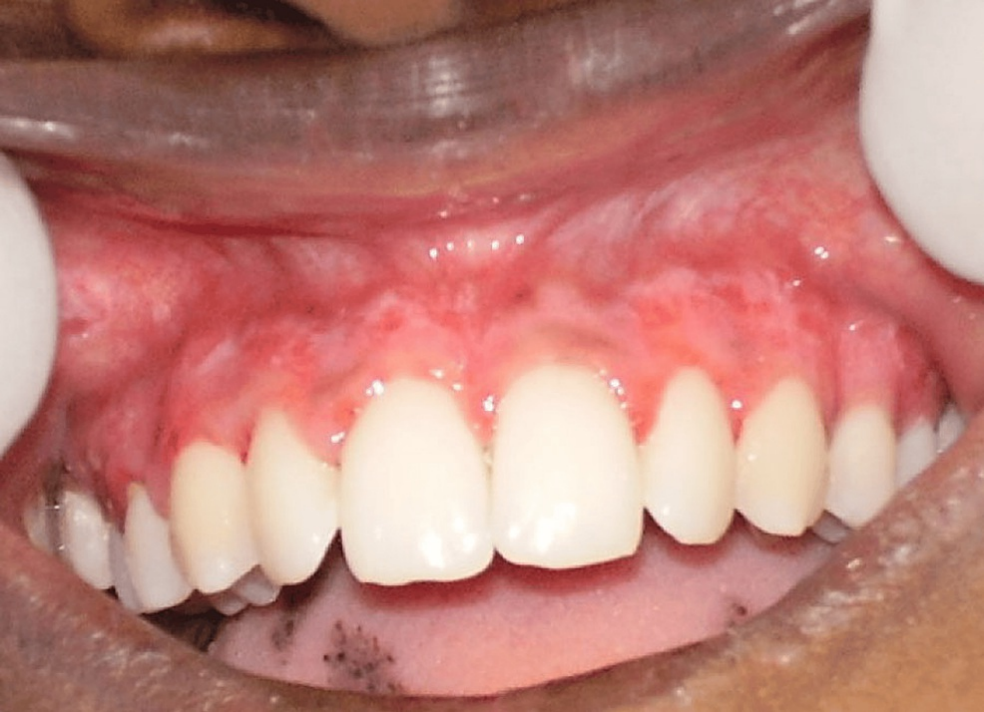
Case 2 - 10 days post-operative. The recovery went smoothly with no post-surgical complications encountered. Minor, pinpoint bleeding spots and erythematous gingiva were observed as the patient did not receive vitamin C.

The periodontal pack was removed after 10 days of surgery, and the treated site was examined. Figures 3-4 depict the postoperative results of the treated site after 10 days. The recovery went smoothly, with no post-surgical complications encountered. We saw that the patient who received vitamin C application showed satisfactory healing with an almost pink gingiva that had no evidence of bleeding or erythema. Minor, pinpoint bleeding spots and erythematous gingiva were observed in the patients who did not receive vitamin C.

## Discussion

Gingival hyperpigmentation is a purplish, diffuse dark brown to black discoloration on the gingiva. Melanin pigment is deposited on the gingiva by active melanocytes, which are dendritic cells that are present in the basal and spinous cell layers of the gingival epithelium. Tyrosinase is an enzyme that plays a major role in the generation of melanin as dopaquinone. Oxidation of tyrosine leads to the generation of the melanin precursor [8]. These pigmentations can be removed by various surgical techniques for aesthetic reasons. Numerous treatment modalities have been employed for depigmentation. Surgical gingival depigmentation techniques are scalpel surgical technique, electrosurgery, cryosurgery, bur abrasion method, lasers, and radiosurgery. Chemical methods that are employed to cover up the gingival pigmentation are acellular dermal matrix allograft and free gingival graft [9].

The method of treatment for gingival depigmentation can be selected based on professional experience and requirements, the patient's financial situation, and personal preferences. This case report is an attempt to study the effects of treating gingival hyperpigmentation by split-thickness gingival excision followed by topical application of vitamin C. The intensity of the gingival pigmentation was assessed using the Dummett Oral Pigmentation Index (DOPI). DOPI was scored as score 1: no clinical pigmentation (pink gingiva), score 2: mild clinical pigmentation (mild light brown colour), score 3: moderate clinical pigmentation (medium brown or mixed pink and brown), and score 4: heavy clinical pigmentation (deep brown or bluish-black) [10].

Melanin pigmentation is removed by de-epithelialization of the superficial layer of gingival epithelium, accompanied by a layer of connective tissue. This leads to an aesthetic transformation of the gingival colour from brown to coral pink. The removal of the superficial layer eventually heals by secondary intention, and a new layer of epithelium is formed that lacks melanin deposits [9].

Vitamin C is an antioxidant that is applied topically to enhance the rate of healing following surgical de-epithelialization. The use of vitamin C shows favourable results in terms of healing. It has a strong antioxidant activity that allows it to inactivate reactive oxygen species (ROS) that damage the structure and function of gingival tissues [11]. ROS are generated by leukocytes during the inflammation process in order to destroy the foreign body. Vitamin C also promotes the synthesis of collagen. It also provides tensile strength to newly formed collagen, which would otherwise be unable to stretch without tearing [12]. Besides its known role in synthesising collagen, research suggests that vitamin C plays a part in enhancing the growth of fibroblasts, which is crucial for the process of wound healing [13]. As vitamin C is utilised during these functions, there is an indication of heightened utilisation at wound sites, implying that supplementing this vitamin might be advantageous in the healing process. Furthermore, inflammation is likely to accelerate the breakdown and use of vitamin C. Inflammation is commonly present to some extent in all wounds and tends to increase in infected areas [14].

Dummett and Barens studied the effects of locally injecting vitamin C for oral mesotherapy. It is a minimally invasive, non-surgical, and efficient technique for gingival depigmentation. Patients in this study were satisfied with the results of the gingival colour obtained and the overall treatment experience [15]. Chaudhary et al. compared gingival depigmentation, pain scores, and itching with a scalpel technique and a nonsurgical intramucosal vitamin C injection. It was found that vitamin C mesotherapy showed better post-operative results than the scalpel technique in the reduction of areas and intensity of gingival hyperpigmentation [16]. Various case reports and reviews of the literature have given positive feedback on the application of vitamin C for gingival depigmentation procedures [17-19].

In this study, the surgical depigmentation of gingiva along with the use of topical vitamin C showed satisfactory results in 10 days, followed by gingival colour and overall patient comfort. The patient presented better healing with an almost pink gingiva with no traces of bleeding or erythema. On the other hand, we saw that without vitamin C, the surgical site showed numerous bleeding spots and erythematous gingiva.

## Conclusions

Gingival hyperpigmentation has been an aesthetic concern among people. Various techniques have been incorporated for gingival depigmentation. The results obtained with the present surgical technique were satisfactory and provided substantial benefits to the patient. The difference in healing rates between the two cases suggests positive effects of vitamin C in the healing phase of gingival tissues. However, larger sample sizes and long-term follow-ups help to prove the importance of ascorbic acid in accelerating the rate of healing of the gingiva after gingival depigmentation has been performed.
